# Developing an Ecotoxicological Classification for Frequently Used Drugs in Primary Care

**DOI:** 10.3390/ijerph22020290

**Published:** 2025-02-16

**Authors:** Tiphaine Charmillot, Nathalie Chèvre, Nicolas Senn

**Affiliations:** 1 Department of Family Medicine, Unisanté, University of Lausanne, 1015 Lausanne, Switzerland; nicolas.senn@unisante.ch; 2 Faculty of Geosciences and Environment, University of Lausanne, 1015 Lausanne, Switzerland; nathalie.chevre@unil.ch

**Keywords:** ecotoxicology, primary care, surface water, drug prescribing, API

## Abstract

Most drugs excreted in urine are not filtered by wastewater treatment plants and end up in aquatic systems. At concentrations measured in waters, toxic effects on species have been described. Second, most of the drug consumption is attributable to primary care prescriptions. We thus present here, an ecotoxicity classification of the most sold drugs in primary care in Switzerland. Three datasets were combined: (1) surveyed ecotoxic drugs by the Swiss National Surface Water Quality Monitoring Programme and its European equivalent, (2) the top 50 drugs by sale in primary care in Switzerland, and (3) active pharmaceutical ingredient (API) concentrations in Lake Geneva and the rivers of the canton of Vaud between 2017 and 2022. We classified APIs into five categories from the safest to the least safe: (1) APIs found in concentrations (C) <10× their environmental quality standard (EQS·10^−1^), (2) EQS·10^−1^ < C < EQS and not listed by the Swiss or the EU Watch List, (3) EQS·10^−1^ < C < EQS and listed, (4) C > EQS and not listed, and (5) C > EQS and listed. We obtained full ecotoxicological data for 35 APIs. Fifteen APIs were designated as safe (category (1):paracetamol, tramadol, amisulpride, citalopram, mirtazapine, metformin, gabapentin, lamotrigine, primidone, candesartan, irbesartan, atenolol, hydrochlorothiazide, ofloxacin, sulfadiazine), eleven as intermediately safe, and nine were of concern (azithromycin, ciprofloxacin, clarithromycin, sulfamethoxazole, carbamazepine, diclofenac, ibuprofen, iomeprol, iopromide). Full data were available for only one-third of the drugs most sold in primary care. Where data do exist, we observed significant differences in environmental impact among the same class of drugs. Our classification could therefore help guide doctors to adopt more eco-friendly prescriptions.

## 1. Introduction

In 2022, a large meta-analysis showed that pharmaceutical compounds are polluting the surface waters of all five continents [1]. Pharmaceuticals reach wastewater treatment plants (WWTPs) after being excreted by patients in urine and faeces or released from drug manufacturing plants. In WWTPs, the common purification techniques are not designed to remove pharmaceuticals [2], therefore a high number of them enter aquatic systems and can accumulate in lakes. In a 2021 scoping review, not less than 286 different active pharmaceutical ingredients (APIs) were found in 260 lakes from 44 countries around the world [3]. Two-thirds exceeded 0.05 μg/L (the Swiss maximum safety threshold = 0.1 μg/L [4]). Switzerland is no exception, with around 30% of micropollutants escaping WWTP filtration [5].

The continuous discharge of active pharmaceutical ingredients (APIs) from WWTP effluent to surface water is of high concern. Indeed, depending on the concentration in wastewater, a wide range of adverse effects have been described on fauna and flora. For example, carbamazepine was shown to impair chloroplast development in algae, cause mutations in mussel mRNA or oxidative stress in rainbow trout [6,7,8]. Several adverse effects were demonstrated in fish, such as 1 μg/L of diclofenac inducing acute tubular necrosis, 0.5 μg/L of propranolol is embryotoxic, and only a few nanograms/L of fluoxetine or sertraline can cause adverse morphological changes in reproductive organs, while fluoroquinolones affect the feeding behaviour of fish and birds [3,6,7,8]. Furthermore, it was demonstrated that benzodiazepines and gemfibrozil inhibit algae and vegetable growth [8,9].

In addition to directly harming the ecosystems in which they infiltrate, APIs can bioaccumulate in the food chain and reach remarkably high concentrations in predatory animals. For instance, an Australian study examined API concentrations in aquatic invertebrates from six streams receiving treated sewage water in Melbourne [10], and as the platypus’ diet is based on aquatic invertebrates, the authors estimated that a platypus ingests up to 2 mg of pharmaceuticals daily.

Another aspect of pharmaceutical micropollution is the so-called cocktail effect. Studies have demonstrated the adverse effect of specific APIs to specific species, while the cumulative effect of lower doses of several APIs is less studied, and this effect should not be underestimated. Indeed, a Canadian study demonstrated that a drug-mix containing 0.5 μg/L of paracetamol, carbamazepine, gemfibrozil, and venlafaxine severely altered proximal kidney tubule morphology in zebrafish [11].

In contrast to the effects of APIs on biodiversity, the impact on humans through drinking water has been hardly investigated, although the few existing studies indicate that they may represent a serious risk to human health. At present, the most studied effect is antibiotic resistance. WWTPs do not filter out antibiotics and antibiotic-resistant pathogens completely, which end up in water and soil where they seriously jeopardise drinking water quality [12]. In addition, antibiotic-resistant bacteria can increase local resistance in the environment via antibiotic-resistant gene transfer [13].

Further, direct adverse API effects have been described in humans such as immunosuppression, genotoxicity, carcinogenesis, allergy, and teratogenicity, with children seeming to be at greater risk than adults [14].

While major impacts of APIs on the environment are known, API emission into wastewater is currently not subject to global regulation. The European drug manufacturing regulation from 2006 states that each new API must be assessed for its environmental effect [15]. However, this applies to acute effects only and not to long-term problems, which limits its overall relevance [12]. Moreover, this regulation only applies to APIs commercialized after 2006 [12]. Finally, even if a newly developed API demonstrates high ecotoxicity, this does not prevent its marketing in human medicine, unlike in veterinary medicine [16]. This lack of guidance contrasts with other micropollutants like pesticides, which have been regulated in Switzerland for more than 25 years [17].

In addition to their direct ecotoxicological impact, pharmaceuticals contribute to other environmental degradation such as via greenhouse emissions. According to a study published in The Lancet, pharmaceuticals and chemicals contributed to 20–32% of the total greenhouse gas emissions of the English national health system in 2019 [18]. Furthermore, drug prescribing is estimated to account for up to 60% of a general practitioner’s (GP’s) carbon footprint, mostly because of metered dose inhalers [19,20]. Therefore, GPs, through their prescriptions, play a vital role in terms of drug-related environmental impact.

In this respect, the development of recommendations that include the environmental impact of common drugs to promote better and more environment-friendly prescriptions is a necessity [21]. Indeed, such guidelines were demonstrated to significantly influence physician decision-making and effectively reduce drug-related pollution [22]. Moreover, it has also been shown that patients are interested in including environmental consideration in their medical treatment, especially for minor ailments and chronic conditions [23]. Despite the potential environmental benefit of environmentally directed recommendations, no ecological prescription guidelines for physicians combining prescribing habits (volumes), environmental toxicity, and the current water concentrations exist.

In the present study, we propose a comprehensive ecotoxicological classification of drugs that includes those on the list of the 50 most sold drugs (in kg) in primary care (GPs, paediatricians, frequently sold over-the-counter drugs) in Switzerland. This classification should allow physicians to consider ecotoxicity in their daily practice. Furthermore, identifying the most sold drugs in primary care will help to better target monitoring of the pharmaceutical pollution of water. The word “API” will be is used in this paper, but we are aware that in the medical field, the term “drug” is more common. Therefore, both terms are considered equivalent in this interdisciplinary paper.

## 2. Materials and Methods

### 2.1. Study Design

This study is a collaboration between the Faculty of Geosciences and Environment of the University of Lausanne (UNIL) and the Department of Family Medicine of the Centre of General Practice and Public Health of Lausanne (Unisanté).

To develop a comprehensive classification of the ecotoxicity of drugs frequently used in primary care in Switzerland, we performed a data analysis on three complementary sources of information: (1) a dataset of the concentrations of active pharmaceutical ingredients (APIs) in the surface waters of the canton of Vaud, (2) the ecotoxicological potential of APIs (based on the published literature/databases), and (3) the top 50 most sold drugs by GPs in Switzerland.

### 2.2. Settings

This study took place in the canton of Vaud, which is bordered by the Lake of Geneva (surface area of 580 km^2^) to the south, which makes this canton of high interest for aquatic ecotoxicity. We focused on primary care as it represents the major origin of drug prescription/utilization [18].

### 2.3. Data Sources

#### 2.3.1. Analysis of API Concentrations in Surface Waters

The canton of Vaud has established a survey of twenty rivers and the Commission Internationale pour la protection des eaux du Léman (CIPEL) has been monitoring Lake Geneva for decades.

Micropollutants in Lake Geneva have been analysed twice a year by CIPEL since 2004. We focused on measurements from 2020 to 2021, where 73 APIs were monitored. Water samples are analysed by the SCITEC Research Laboratory SA in Lausanne using the liquid chromatography–mass spectrometry technique (LCMS/MS) [24].

The rivers of the canton of Vaud are monitored twelve times a year by the Direction de l’environnement industriel, urbain et rural (DGE-DIREV), a sector of the Department of the Environment of the canton of Vaud [25]. The samples are analysed by the laboratory of the DGE itself using the LCMS/MS technique [25]. We obtained their raw data from 2017 to 2022, where 50 APIs were monitored [26].

Once we extracted all the data, we classified data by API and for each, we identified the highest measured water concentration. To evaluate the possible toxicity of the measured concentrations, the Predicted No-Effect Concentration (PNEC), or ecotoxic threshold, is reported for each substance. We used PNECs approved by the Ecotox Centre, the centre for applied ecotoxicology in Switzerland that provides PNEC values for 44 APIs [4]. For missing antibiotics, we found PNEC values in the list of AMR Industry Alliance, which contains PNECs of 126 antibiotics [27]. Additionally, we found the rest of the PNECs in the NORMAN Substance Database [28]. When differing PNECs were available for the same API, we chose Ecotox’s PNEC because they are established and are stronger values than NORMAN’s or AMR’s. However, for antibiotics where AMR suggested a lower PNEC considering the minimum inhibitory concentration (MIC), we chose this one to take antibiotic resistance into account (as it is not the case in the Ecotox database). Then, we classified substances according to whether their concentration in surface water exceeded their respective PNEC or not.

Although the definition of EQS (environment quality standard) differs slightly from PNEC in that the former is the standard term in the law and in water policies, we consider both terms identical here.

#### 2.3.2. Selection of the Most Ecotoxic APIs

In Switzerland, micropollutants in surface water are surveyed by the National Surface Water Quality Monitoring Programme (NAWA). This program is a collaboration between the Federal Office for the Environment and the cantons. It monitors 72 micropollutants at 38 sites across the country, among which 46 are APIs [25,29].

The first EU Watch List was established in 2013 to monitor and improve available information on potentially harmful substances. If sufficient evidence is obtained, the substance of concern is added to the Priority Substance List, and EU member states have to monitor them to ensure that they do not exceed their established EQS in surface water. Since then, the Watch List has been updated every second year [30].

In this work, we considered the latest Watch List (2022) of 26 substances containing 10 APIs (clindamycin, ofloxacin, sulfamethoxazole, trimethoprim, guanylurea, metformin, venlafaxine, clotrimazole, fluconazole, and miconazole).

To understand the API inclusion criteria in the EU Watch List, we contacted the Ecotox Centre, the Swiss centre collaborating on the elaboration of this list, and decided to include the complete list.

#### 2.3.3. Selection of the Top 50 Most Sold Drugs in Primary Care in Switzerland

To obtain the list of most sold drugs in primary care in Switzerland, we contacted three pharmaceutical statistics companies: Interpharma, the association of Switzerland’s research-based pharmaceutical industry, Pharmasuisse, the Swiss society of pharmacists, and SASIS AG, the umbrella association of Swiss health insurers. After a long and tedious process, we obtained data against an access fee from SASIS AG.

The inclusion criteria for this dataset included all drugs sold directly at the GP’s office or in pharmacies on prescription from GPs and paediatricians in Switzerland from 2020 to 2022. Additionally, we asked for the inclusion of the over-the-counter sales of ibuprofen, diclofenac, acetylsalicylic acid, naproxen, and paracetamol in the same period, as they represent a large proportion of drug sales.

SASIS AG provided us with an Excel file containing the drugs sold in each canton, classified by trade name and quantity of active substance in mg or μg. For each item, the number of dosage forms in the box and the international non-proprietary name (INN) were specified (e.g., DAFALGAN, tablet 500 mg, 16 pieces, paracetamol). Data were extracted and processed using the R software (R 4.2.1). We began by separating the composite drugs to obtain the quantity of each substance. Units had to be harmonized, and all concentrations were transformed into mg. The quantities of each substance in trade name were obtained by multiplying the number of the galenic form by the respective concentration. We then combined the different brands of the same substances to obtain the total quantities for the 1300 INN. These were then converted into kg and ranked in descending order (Table 1). As data were obtained in the trade name and not INN, and as concentrations were in different units, numerous approximations had to be made when calculating the final quantities.

### 2.4. Classification

At the end of the data collection, we compiled the data in a table (Table 2). We grouped each API by class and for each API, we recorded, when available, the following information: (i) its EQS, (ii) the highest concentration measured by CIPEL and DIREV, and (iii) whether the API featured in the top 50 most sold drugs list. We highlighted in red the APIs listed on the NAWA or EU Watch List.

We only kept APIs for which the EQS was known, and monitoring was performed by at least one organisation (CIPEL or DIREV). Then, we evaluated the effective ecotoxicity risk of each API by dividing its maximum concentration by its EQS. Additionally, as per ecotoxicological protocols, we established the 10× security factor from the EQS for an API to be considered safe. Finally, we established an ecotoxic API classification from the lowest to the highest risk based on the combination of these different types of information.

Thus, our ecotoxicity classification has five categories (from the lowest to the highest risk to the environment) and a sixth category indicating APIs with incomplete/no data:

1. APIs with concentrations in Lake Geneva and the rivers of the canton of Vaud lower than 10x their own EQS (EQS·10^−1^).
2. APIs with concentrations higher than their EQS·10^−1^ but lower than EQS.
3. APIs with concentrations higher than EQS·10^−1^ but lower than EQS and that are listed in the NAWA or in the EU Watch List.
4. APIs with concentrations higher than their EQS.
5. APIs with concentrations higher than their EQS and that are listed in the NAWA or in the EU Watch List.
6. APIs with incomplete data (measurement or EQS), but which are in the top 50 most sold drugs in primary care in Switzerland.

The last category (n°6) is not part of the classification itself but highlights the lack of information about commonly used APIs.

### 2.5. Ethics

This study is a data analysis of non-human or animal data. Thus, its protocol did not require ethical approval according to Swiss regulations.

## 3. Results

### 3.1. Concentration of APIs in the Surface Water of the Canton of Vaud

In 2020–2021, APIs were detected in the waters of Lake Geneva but never exceeded their corresponding EQS [24]. The most concentrated API was metformin (highest concentration 0.5 μg/L), followed by methenamine (0.1 μg/L), carisoprodol (0.04 μg/L), carbamazepine (0.02 μg/L), and mepivacaine (0.03 μg/L). In total, the annual load of pharmaceutical residues in Lake Geneva was estimated at 6314 kg in 2021 (6447 kg in 2020).

Regarding the rivers in the canton of Vaud, we obtained data from 2017 to 2022, where 35 APIs were monitored in 20 rivers [25,26]. Among these, 9 APIs exceeded their threshold at least once (without security factor): iomeprol, 35% above threshold; iopromide, 29%; ibuprofen, 26%; azithromycin, 19%; diclofenac, 12%; ciprofloxacin, 5%; clarithromycin, 1.3%; sulfamethoxazole, 0.4%; and carbamazepine, 0.2%. The highest concentrations were reported for iomeprol (28.1 μg/L), metformin (16.1 μg/L), gabapentin (10.4 μg/L), diclofenac (6.2 μg/L), and irbesartan (3.9 μg/L) [26].

Additionally, in the canton of Vaud, the most concentrated pharmaceuticals in wastewater treatment plant (WWTP) effluents were metformin, gabapentin, irbesartan, hydrochlorothiazide, and diclofenac, all exceeding 1 μg/L (concentration range 2.4 to 66.1 μg/L) [5]. For most APIs, the concentration in the WWTP effluent water is close to the concentration in the influent water, indicating poor elimination by the filtration system.

### 3.2. Analysis of the Most Ecotoxic APIs

#### Swiss Legislation

The API inclusion criteria in the NAWA Watch List are either high ecotoxic potentials (high EQSs or PNEC values) or high prescription rates. Additionally, some APIs used to be monitored by one or several cantons before the creation of the NAWA and thus remained on the list. Hence, we contacted NAWA and found that among the 46 listed components, 7 were on the list because of their ecotoxicity (EQS lower than the Swiss threshold = EQS < 0.1 μg/L, except 0.05 for diclofenac and 0.019 for azithromycin) [31], including ibuprofen, diclofenac, ciprofloxacin, azithromycin, sertraline, 17-beta-estradiol, and 17-alpha-ethinylestradiol.

As the EQSs of 11 other APIs on the list had not been validated by NAWA, we included them in our list as potentially ecotoxic APIs, including hydrochlorothiazide, venlafaxine, amisulpride, citalopram, tramadol, levetiracetam, gabapentin, lamotrigine, sotalol, iopamidol, and iopromide.

### 3.3. Analysis of Most Sold Drugs in Switzerland

Table 1 shows the top 50 most sold drugs in Switzerland, prescribed by GPs and paediatricians.

### 3.4. A Classification System

**Figure 1:**
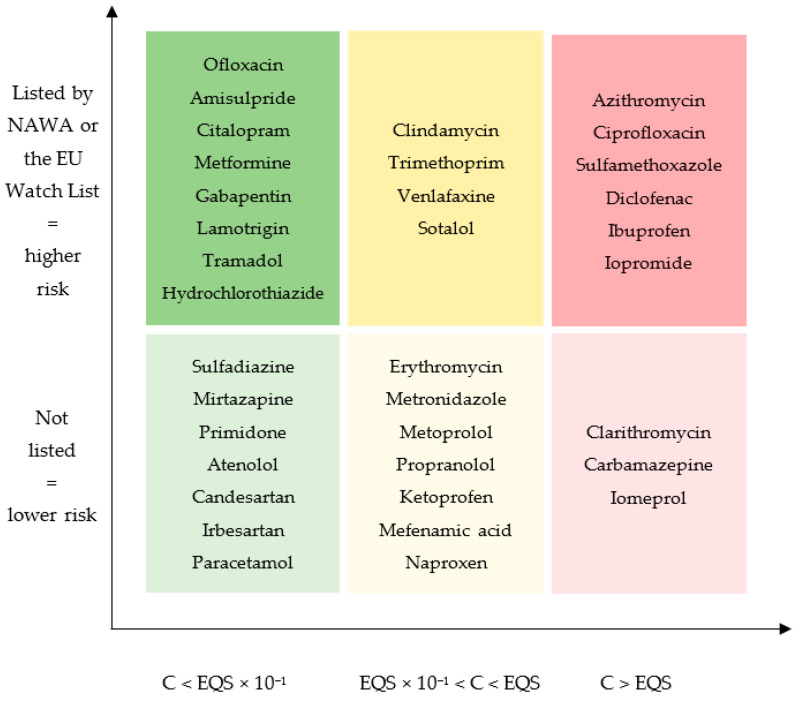
Ecotoxic classification of most sold drugs in primary care in Switzerland (Author: T. Charmillot, 2025). This classification is based on API concentrations effectively measured in the environment related to their individual EQSs (x-axis) and whether they are classified by the toxic lists (NAWA or EU) (y-axis). NAWA: Nationale Beobachtung Oberflächengewässerqualität; C: concentration; EQS: environmental quality standards; EU: Europe.

Table 2 contains 53 APIs. Of these, 28 have an established EQS by the *Ecotox Centre*, which guarantees the highest reliability. The 25 others come from the AMR or NORMAN databases. A total of 18 APIs were only monitored in the lake and not in the river. As we found lower concentrations in the lake than in the rivers, we decided that the absence of monitoring in the rivers was an exclusion criterion for an API to be further classified. Thus, 35 APIs could be used for the ecotoxic classification shown in Table 3. Figure 1 presents the same results, showing the risk rates (low, medium, high) using a color code.

Antibiotics is the category with the most information available, providing a more reliable comparison. A few of them are labelled safe for surface water (lower risk), including ofloxacin, sulfadiazine, erythromycin, metronidazole, clindamycin, and trimethoprim, because they were found to have concentrations lower than their respective EQSs. In contrast, clarithromycin, azithromycin, ciprofloxacin, and sulfamethoxazole have lower EQSs and were found in high concentrations.

For non-steroidal anti-inflammatory drugs (NSAIDs) and painkillers, paracetamol and tramadol were shown to have lower risk than ibuprofen and diclofenac, which have 100 times higher toxicity levels (EQS: 46, 8.9 vs. 0.01, 0.05 μg/L). Ketoprofen, mefenamic acid, and naproxen showed an intermediate risk.

Among antihypertensives and beta-blockers, atenolol, candesartan, and irbesartan were the safest molecules. Atenolol is to be preferred to metoprolol, propranolol or sotalol from an ecotoxicological perspective. Hence, metoprolol, propranolol, and sotalol do not comply with our precautionary principle, under which the maximum measured concentration must be 10 times lower than the respective EQS (metoprolol: max concentration 1.1, EQS 8.6 μg/L; propranolol: max concentration 0.09, EQS 0.16 μg/L; sotalol: max concentration 0.8, EQS 6.5 μg/L).

Among diuretics, only hydrochlorothiazide could be classified, as other diuretics were not monitored in surface water, and was classified in the lowest-risk category.

Amisulpride, citalopram, and mirtazapine were the lower risk molecules among the antidepressants for which we have data. Venlafaxine is the second-best choice (max concentration 0.55 μg/L, EQS 0.88 μg/L) as it shows a higher ecotoxicological risk (category 3 vs. 1 for the three above-mentioned drugs).

In the antiepileptic class, gabapentin, lamotrigine, and primidone were the APIs with the lowest risk (cat. 1). By contrast, carbamazepine was measured at concentrations exceeding its EQS (2.3 vs. 2 μg/L, cat. 4).

Finally, iodinated contrast agents were the most problematic drug class, as the only two classifiable APIs, iomeprol and iopromide, both exceeded their EQSs in 30 to 40% of measurements.

Among the 35 classified APIs, 16 are in the top 50 most sold drugs. All except ofloxacin, sulfadiazine, and erythromycin are in the top 200 most sold drugs in primary care, out of a total of 1300 drugs (in international non-proprietary names).

For several API classes listed in the top 50 most sold drugs, we could not identify a “lower-risk” option because of limited data availability; either no EQS was established, or the APIs were not monitored in the surface waters of the canton of Vaud. This is the case for antacids, antiplatelets, lipid-lowering agents, antiarrhythmics, anaesthetics, neuroleptics, antivirals, spasmolytics, veinotonics, and hypouricemics.

Finally, we excluded hormones from this table since the detection threshold is higher than their EQSs (0.001 vs. 0.0005 μg/L).

### 3.5. Comparison with API Concentrations in Surface Waters of Europe

To determine if the results of this study are generalisable, we compared API concentrations in the surface waters of the canton of Vaud with two international studies. Among other studies, these were chosen because of their comparable research field, sampling methodology, and recentness. First, a Scottish study conducted in 2021 where samples were taken all along the river Dee over one year [32]. Eight APIs were analysed: paracetamol, ibuprofen, diclofenac, clarithromycin, trimethoprim, carbamazepine, fluoxetine, and 17α-ethynyloestradiol.

In this sampling, the highest concentrations were seen with gabapentin, valsartan, ciprofloxacin, diclofenac, venlafaxine, and paracetamol. Five APIs were constantly detected: ibuprofen, paracetamol, trimethoprim, diclofenac, and carbamazepine (Table 4).

In the second study, 78 APIs were measured in the river Mijares (Spain) for nine months in 2019 [33]. The highest concentrations were seen for phenazone, tramadol, gabapentin, irbesartan, valsartan, azithromycin, and ciprofloxacin (Table 4).

The comparisons indicated that there is a 10 to 100x dilution factor for lake measurements compared to rivers. APIs found in the rivers of the canton of Vaud are similar to those found in other European rivers but tend to be more concentrated. The size of the chosen rivers (smaller in Switzerland than in Scotland or Spain) or the population density in the catchment area could be possible explanations. Gabapentin is the only molecule found in the top five substances in all three rivers.

## 4. Discussion

The current study provides an ecotoxicity classification of APIs, including those frequently prescribed in primary care in Switzerland. After summarising the data, individual surface water concentrations and EQSs were available for 35 APIs. Out of these, 16 are part of the top 50 most sold drugs in primary care. This enabled us to compile an API classification from lowest to highest risk. We observed that when all API information is available, substantial differences exist in terms of ecotoxicity, even within the same drug class. We also found out that for many drugs sold in primary care, we have insufficient information to estimate their risk to the aquatic environment. As a reminder, this study focuses only on the effect of stand-alone molecules and does not consider their potential effects at lower concentrations, imputable to mixture or concentration addition. We decided to adopt a worst-case scenario approach and thus chose the maximum concentration in surface water, where other researchers could have chosen the average concentration. In addition to the safety it guarantees, this choice allows us to consider the fact that these substances are emitted continuously, as well as consider seasonality and external events (e.g., the COVID-19 pandemic). We therefore believe that this choice provides a high level of safety, especially for molecules identified as “low risk”.

Additionally, we observed that a high API concentration alone in surface water does not necessarily imply a risk of ecotoxicity, based on the current available ecotoxicity data. Hence, metformin, irbesartan, and gabapentin are among the most concentrated monitored molecules, but as they have a high EQS, they feature among the safest APIs in the classification. Similarly, a high sale rate or low EQS is not a reliable criterion on its own to assess a molecule’s ecotoxicity. This emphasises the need for integrative studies.

### 4.1. Using API Classification in Primary Care

Our classification aims to provide pragmatic information to physicians on the ecotoxicological effect of the drugs they prescribe. It aims also to be integrated more globally into the management plans of patients with proposals for drug prescription strategies (contribute to choose the drug with the lowest environmental impact), either for new treatment introductions or when reviewing long-term treatment plans. This “environmental prescription” is to be considered as another decision factor, concomitantly with the patient’s comorbidities, safety of use, evidence-based literature, costs, etc.

**Figure 2:**
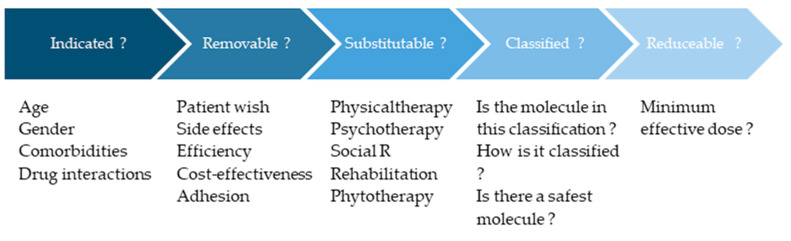
Prescription process integrating the ecotoxicological classification.

The classification may be used as part of a prescription strategy that should include the following steps (Figure 2): (1) reconsidering the drug indication and if it is safe for the patient’s profile; (2) evaluating whether a drug can be removed because of patient preference, absence of evidence-based efficacy for a specific condition, etc; (3) assessing non-pharmaceutical therapeutic options; (4) in the absence of a suitable alternative therapy, and if a drug prescription is needed, then consider the ecotoxicity classification to choose the drug with the lowest environmental impact, if applicable; and (5) regardless of the drug prescribed, the lowest effective dose must be prescribed. This strategy is well aligned with Smarter medicine guidelines provided by Choosing wisely Switzerland (URL https://www.smartermedicine.ch/de/home, accessed on 26 June 2024).

### 4.2. Priority List Comparisons

Some countries have developed their own comprehensive guidelines relative to the European priority substances list. Sweden for example, has its own “Table of environmentally hazardous drug substances” for the Stockholm region [34] and is a pioneer country in environmentally sustainable prescribing, as it has been addressing this issue for 25 years [35]. Additionally, Stockholm has already had its own prescription guidelines since 2016 and is, to our knowledge, the only country to have done so (URL https://www.janusinfo.se, accessed on 3 March 2022). Venlafaxine is the only drug found on all three lists (Swedish, EU, and NAWA). The molecules found in both the NAWA and the Swedish priority lists are diclofenac, azithromycin, ciprofloxacin, sertraline, and oestradiol.

### 4.3. Limitations

Lack of data: We could only classify one-third of the fifty most sold drugs. Hence, for most APIs, the EQS either does not exist in the literature or has not been validated. In addition, there is no international reference database. Furthermore, some EQS lists—for example, the French reference database—identify PNECs for various pollutants but not for drugs [36]. This lack of unified international monitoring is worrying and hinders ecotoxicity research work.

Data access: In addition to the lack of API data, access to data was also a challenge. While CIPEL and DIREV immediately agreed to share their data, obtaining access to drug sale data was extremely difficult. While drug data exist (volume of drugs sold, etc.), they are often not shared, and if access is possible, it is subject to high fees and restrictive rules, making independent academic research difficult. Moreover, the received data were scattered and not provided in a user-friendly format.

The difficult access to drug data in Switzerland is thus highly problematic, as it discourages or even prevents the development of studies on drug consumption and makes it hard for public health services and the population to obtain reliable, independent information. Furthermore, it is questionable that data highly relevant for public health and safety are not accessible. Interestingly, this limitation seems to be specific to Switzerland (an unregulated market-driven healthcare system), as, for example, drug consumption data are openly published and free of charge in Sweden [22].

Data collection period: We are aware that the pandemic may have slightly changed the usual drug consumption/prescription habits. However, we believe that CIPEL’s long period of observation (2 years), the fact that the kind of drugs prescribed is very stable over time, the multi-database approach, and the limited lockdown conducted in Switzerland during the pandemic reduce the possible effects of the pandemic on our results.

### 4.4. Innovation and Interdisciplinarity

To our knowledge, this is the first study combining ecotoxicity, concentration in surface water, and prescription volumes in Switzerland. This “environmental impact” is to be considered as another factor influencing the prescription decision-making process.

### 4.5. External Validity of the Methodology

This study confers highly relevant data for the canton of Vaud and the western part of Switzerland. Furthermore, the classification was developed to enable any other region of Switzerland or even other countries to propose a standardised methodology for classification in its setting. Hence, as monitoring surface water is mandatory, local API water concentrations can be replaced in the data summarising table (Table 2), and EQSs compared with concentrations and sales to derive a local and tangible classification.

### 4.6. Interpretation of the Results for Healthcare Professionals

Even though hospitals, drug manufacturers, and agricultural practices release APIs into the wastewater, the leading cause of drug pollution in Switzerland is via human consumption [1]. Furthermore, measurements of the sewage in the canton of Vaud showed that domestic drug consumption influences API concentrations in surface water more than hospitals or nursing homes do [5]. Consequently, pharmaceutical micropollution relates first to the population density served by WWTPs and second to the disease burden in the population, public access to healthcare, and domestic drug consumption patterns [1]. Therefore, doctors and pharmacists play a key role in preventing this pollution.

For this purpose, we developed an ecotoxicological classification based on existing data. As stated, the data are currently incomplete, hindering an exhaustive comparison. Moreover, data scarcity leads to a pre-selection bias in subsequent studies, the so-called Matthew effect [37]. This was observed in the literature review; among the scarce literature available on drug ecotoxicity, a considerable proportion focuses on NSAIDs and antibiotics, especially diclofenac and ciprofloxacin. Consequently, research must be strengthened to provide EQSs for a wider range of APIs and monitoring must be related to consumption data, excretion profiles, removal by WWTPs, and degradation in the environment. Simultaneously, WWTP elimination technology improvements, strengthening collaboration with veterinary medicine on drug use and control, and tightening the control of emissions released by the manufacturing industry are additional measures to reduce API concentrations in surface water.

Furthermore, the classification table must be integrated into wider prescription guidelines. Evidence-based prescribing, de-prescribing, and non-pharmaceutical therapies should become central objectives in primary care. Other possibilities to reduce waste are to ask patients what medication they already have at home before prescribing new ones or providing a limited number of tablets or starter packs for new drugs or using the patient’s own drugs during hospitalisation. The end life of drugs should also be considered, as studies demonstrated that expired drugs were still active even when storage was suboptimal [38]. This finding calls for a re-assessment of the expiration dates and an introduction of a collection system for expired medicines [21]. Simultaneously, drug ecotoxicity must be thematised among healthcare professionals as well as with patients to develop a collaborative therapeutic strategy.

Finally, climate change has been shown to be an additional factor aggravating pharmaceutical micropollution [7]. The climate crisis is increasing the magnitude and occurrence of extreme weather events. As a result, droughts decrease the dilution of APIs in WWTP effluents, while floods induce the remobilisation of micropollutants from river sediments or cause sewer overflows [39]. Therefore, it is essential to increase rational prescribing, WWTP efficiency, and to strengthen interdisciplinary collaboration to increase knowledge about pharmaceutical micropollution.

### 4.7. Interpretation of the Results for Monitoring Programs

As we see, several drug classes from the top 50 most sold drugs could not be classified because of a lack of monitoring. Hence, it would be of high interest to obtain the selection criteria for APIs applied by monitoring programmes. This would enable us to better understand why frequently used drugs in primary care such as amoxicillin, antacids, clopidogrel, lipid-lowering agents or allopurinol are currently not being monitored, and to what extent elimination rate, removal efficiency from WWTPs, analytical challenges, high EQSs or other factors play a role in this selection. This could additionally help in understanding the need for considering elimination rate and filtration efficiency from WWTPs, as opposed to using surface water concentration values alone in subsequent studies.

### 4.8. Generalisability

To evaluate the external validity of our results, we compared the list of the least safe APIs obtained in our classification (C > EQS = categories 4 and 5) with a study conducted in Scotland in 2021. In the Scottish study, the authors developed an analytical methodology to rapidly determine priority pharmaceutical compounds by using five criteria, consumption data, environmental occurrence, wastewater treatment removal efficiency, toxicological effects, and regulatory indicators [40], providing 17 APIs of concern that were validated with measurements. After excluding hormone and roxithromycin for which data are not available in Switzerland, half of the APIs found in our study are similar to the Scottish classification (diclofenac, ibuprofen, clarithromycin, ciprofloxacin, and carbamazepine). In our classification, atorvastatin and fluoxetine were not evaluable (cat. 6) because of missing monitoring information.

As we can see, the most problematic API lists vary from country to country, so classifications need to be adapted to reflect the variability of medical practices and contexts. On the other hand, diclofenac, ibuprofen, clarithromycin, ciprofloxacin, and carbamazepine seem to be priority compounds to monitor in Europe, as they were highlighted as higher risk in both studies. We thus need to harmonise and complete the data in several ways: validate EQSs for frequently sold drugs, systematically monitor in rivers and lakes the concentrations of APIs that feature in the list of top sold drugs, and, finally, better explore the direct ecotoxic effects of frequently used drugs.

## 5. Conclusions

Pharmaceuticals intended to improve health and the well-being of humans end up in aquatic environments where they endanger local ecosystems. Despite the alarming effects on the fauna and flora, no global restrictive regulations are currently implemented. This study revealed that for more than half of the top-selling drugs (in kg) in primary care, the available information is insufficient for providing exhaustive recommendations on their environmental toxicity. Nevertheless, for those with sufficient data, it is possible to establish an ecotoxicological classification that often reveals major differences within the same drug class. Hence, this classification should be seen as an evolving process and regularly re-evaluated to incorporate the latest data.

These results should serve as a model to develop comprehensive ecotoxicological monitoring that also includes the healthcare sector. Indeed, it is essential to increase medical knowledge, to establish EQSs for a wider range of APIs, and to strengthen interdisciplinary collaboration to tackle this present and future environmental issue.

## Figures and Tables

**Table 1:** The top 50 most sold drugs in primary care in Switzerland, SASIS Ag data (Author: R. M. Nasch, 2023).

	API	Total in kg
1	Metformin	35,849
2	Ibuprofen	21,556
3	Paracetamol	20,972
4	Metamizole	11,036
5	Iopromide	9418
6	Acetylsalicylic acid	8154
7	Amoxicillin	4288
8	Iomeprol	3646
9	Mefenamic acid	3235
10	Diosmine	3049
11	Allopurinol	2963
12	Valproic acid	2923
13	Levetiracetam	2881
14	Carbamazepine	2465
15	Pantoprazole	2242
16	Metoprolol	2108
17	Oxcarbazepine	1811
18	Pregabalin	1623
19	Atorvastatin	1516
20	Gabapentin	1353
21	Irbesartan	1283
22	Valsartan	1091
23	Mesalazine	1017
24	Quetiapine	1002
25	Trazodone	985
26	Valaciclovir	829
27	Diclofenac	793
28	Magaldrate	781
29	Iobitridol	750
30	Esomeprazole	732
31	Venlafaxine	672
32	Trimipramine	668
33	Etodolac	664
34	Tramadol	649
35	Clopidogrel	616
36	Lamotrigine	606
37	Ciprofloxacin	543
38	Lithium	536
39	Mebeverine	525
40	Amiodaron	512
41	Naproxen	496
42	Lidocaine	469
43	Iohexol	467
44	Losartan	433
45	Fenofibrate	430
46	Sertraline	426
47	Tolperisone	418
48	Celecoxib	398
49	Gliclazide	397
50	Rosuvastatin	390

SASIS Ag data, Renaud Mika Nasch, 2023. API: active pharmaceutical ingredient; kg: kilograms.

**Table 2:** Data summary table (Author: T. Charmillot, 2025).

	EQS	Measure Max CIPEL (μg/L)	Measure Max DIREV (μg/L)	Top 50 Most Sold Drugs in Switzerland
NSAIDs AND PAINKILLERS				
Diclofenac	0.05 ^1^	<0.01	6.18	X
Ibuprofen	0.011 ^1^	<0.004	0.35	X
Ketoprofen	2.1 ^3^	<0.004	0.22	
Mefenamic acid	1 ^1^	0.002	0.24	X
Naproxen	1.7 ^1^	<0.001	0.63	X
Paracetamol	46 ^1^	0.015	1.72	X
Tramadol	8.86 ^3^	0.004	0.74	X
ANTIBIOTICS				
Amoxicillin	0.078 ^1^	<0.01	-	X
Azithromycin	0.019 ^1^	<0.01	0.44	
Ciprofloxacin	0.06 ^2^	<0.001	0.1	X
Clarithromycin	0.12 ^1^	<0.01	0.44	
Clindamycin	0.1 ^2^	<0.001	0.08	
Erythromycin	0.3 ^1^	-	0.09	
Metronidazole	0.13 ^2^	<0.004	0.08	
Norfloxacin	0.5 ^2^	<0.001	Not measurable	
Ofloxacin	0.5 ^2^	-	0.02	
Sulfadiazine	11 ^2^	-	0.01	
Sulfamethoxazole	0.6 ^1^	0.004	0.72	
Trimethoprim	0.5 ^2^	<0.01	0.35	
ANTIDEPRESSANTS				
Amisulpride	140 ^1^	-	1	
Citalopram	16 ^3^	-	0.22	
Mirtazapine	1 ^3^	<0.004	0.04	
Sertraline	0.009 ^1^	<0.001	-	X
Venlafaxine	0.88 ^1^	<0.01	0.55	X
ANTIDIABETICS				
Guanylurea	100 ^1^	0.3	-	
Metformine	160 ^1^	0.5	16	X
ANTIEPILEPTICS				
Carbamazepine	2 ^1^	0.023	2.2	X
Diazepam	0.29 ^3^	<0.004	-	
Gabapentin	1000 ^1^	-	10.4	X
Lamotrigine	8 ^1^	-	0.7	X
Primidone	9.11 ^3^	<0.004	0.3	
ANTIFUNGALS				
Clotrimazole	0.03 ^3^	<0.01	-	
Fluconazole	1 ^3^	<0.01	-	
Miconazole	0.025 ^3^	<0.01	-	
ANTIHYPERTENSIVES AND BETA-BLOCKERS				
Atenolol	150 ^1^	0.002	0.45	
Candesartan	100 ^1^	-	1.1	
Irbesartan	700 ^1^	<0.004	3.9	X
Metoprolol	8.6 ^1^	<0.004	1.1	X
Propranolol	0.16 ^1^	<0.001	0.09	
Sotalol	6.52 ^3^	-	0.81	
DIURETICS				
Furosemide	31.3 ^3^	<0.004	-	
Hydrochlorothiazide	100 ^1^	-	2.47	
Torsemide	1670 ^1^	<0.001	-	
ANXIOLYTICS				
Lorazepam	0.096 ^3^	<0.001	-	
Oxazepam	0.37 ^3^	<0.001	-	
Zolpidem	0.18 ^3^	<0.001	-	
HORMONES				
Estradiol	0.0004 ^1^	<0.005	-	
Estriol	0.06 ^3^	<0.005	-	
Estron	0.0036 ^1^	<0.005	-	
Ethinylestradiol	0.00004 ^1^	<0.005	-	
NEUROLEPTICS				
Risperidon	0.38 ^3^	<0.001	-	
IODINATED CONTRAST AGENTS				
Iomeprol	0.15 ^3^	-	28	X
Iopromide	0.11 ^3^	-	1.6	X

Red: APIs listed by NAWA or the EU Watch List; DIREV: Direction de l’environnement industriel, urbain et rural; EQS: environmental quality standards; NAWA: National Surface Water Quality Monitoring Programme; NSAID: nonsteroidal anti-inflammatory drugs; ^1^: EQS found in the Ecotox database; ^2^: EQS from AMR; ^3^: EQS from NORMAN.

**Table 3:** Ecotoxicological classification of most sold drugs in primary care in Switzerland (Author: T. Charmillot, 2025).

LOWER RISK		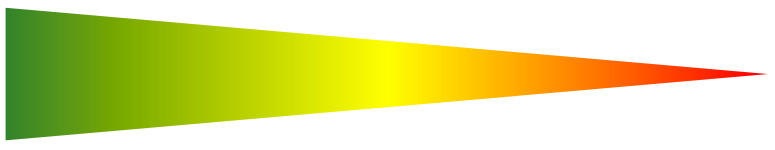					HIGHER RISK
		Category 1 C < EQS × 10^−1^	Category 2 EQS > C > EQS × 10^−1^	Category 3 EQS > C > EQS × 10^−1^ and listed by NAWA or the EU Watch list	Category 4 C > EQS	Category 5 C > EQS and listed by NAWA or the EU Watch list	Category 6 Top 50 most sold drugs, incomplete data
	
ANESTHETICS							**Lidocaine**
ANTACIDS							**Esomeprazole** **Magaldrate** **Pantoprazole**
ANTIARRHYTHMICS							**Amiodarone**
ANTIBIOTICS		Ofloxacin ** Sulfadiazine	Erythromycin Metronidazole	Clindamycin ** Trimethoprim **	Clarithromycin	Azithromycin * Ciprofloxacin * Sulfamethoxazole **	**Amoxicillin**
ANTIDEPRESSANTS		Amisulpride *** Citalopram *** Mirtazapine		Venlafaxine **			Sertraline * **Trazodon** **Trimipramin**
ANTIDIABETICS		Metformin **					**Gliclazid**
ANTIEPILEPTICS		Gabapentin *** Lamotrigin *** Primidone			**Carbamazepine**		Levetiracetam *** **Oxcarbazepine** **Pregabalin** **Valproic acid**
ANTIHYPERTENSIVES AND BETA-BLOCKERS		Atenolol Candesartan **Irbesartan**	**Metoprolol** Propranolol	Sotalol ***			**Losartan** **Valsartan**
NSAIDs and PAINKILLERS		**Paracetamol** Tramadol ***	Ketoprofen **Mefenamic acid Naproxen**			Diclofenac * Ibuprofen *	**Acetylsalicylic acid** **Celecoxib** **Etodolac** **Mesalazin** **Metamizole**
ANTIPLATELETS							**Clopidogrel**
ANTIVIRALS							**Valaciclovir**
DIURETICS		Hydrochlorothiazide ***					
HYPOURICEMICS							**Allopurinol**
IODINATED CONTRAST AGENTS					**Iomeprol**	Iopromide ***	**Iobitridol** **Iohexol**
LIPID-LOWERING AGENTS							**Atorvastatine** **Fenofibrat** **Rosuvastatine**
NEUROLEPTICS							**Lithium** **Quetiapine**
SPASMOLYTICS							**Mebeverin** **Tolperisone**
VENOTONICS							**Diosmine**

* NAWA; ** Watch List EU; *** potentially ecotoxic: EQS not validated but listed by NAWA; **bold**: top 50 most sold in primary care.

**Table 4:** Comparison of top 5 most concentrated APIs in surface waters in Europe and their respective concentrations in rivers.

	River Dee (Scotland)		River Mijares (Spain)		CIPEL (Lake Geneva)		DIREV (Vaud Rivers)	
Top 5 highest concentrations in descending order [μg/L]	Gabapentin	1.9	Phenazone	2	Metformin	0.5	Iomeprol	28.1
	Valsartan	1.6	Tramadol	1.9	Methenamine	0.1	Metformin	16.1
	Ciprofloxacin	1.1	Gabapentin	1.9	Carisoprodol	0.04	Gabapentin	10.4
	Diclofenac	0.9	Irbesartan	1.7	Mepivacaine	0.03	Diclofenac	6.2
	Venlafaxine	0.8	Valsartan	1.6	Carbamazepine	0.02	Irbesartan	3.9
Metformin			-		0.5		16.1	
Irbesartan			1.7				3.9	
Valsartan	1.6		1.6					
Gabapentin	1.9		1.9				10.4	
Diclofenac	0.9		0.9				6.2	
Carbamazepine			0.03		0.02			

DIREV: Direction de l’environnement industriel, urbain et rural; CIPEL: Commission internationale pour la protection des eaux du Léman.

## Data Availability

The datasets used and analysed during the current study are available from the corresponding author on reasonable request.

## References

[1] Adeleye A.S., Xue J., Zhao Y., Taylor A.A., Zenobio J.E., Sun Y., Han Z., Salawu O.A., Zhu Y. (2022). Abundance, fate, and effects of pharmaceuticals and personal care products in aquatic environments. J. Hazard. Mater..

[2] Al Aukidy M., Verlicchi P., Voulvoulis N. (2014). A framework for the assessment of the environmental risk posed by pharmaceuticals originating from hospital effluents. Sci. Total Environ..

[3] Katsikaros A.G., Chrysikopoulos C.V. (2021). Occurrence and distribution of pharmaceuticals and personal care products (PPCPs) detected in lakes around the world—A review. Environ. Adv..

[4] Oekotoxzentrum Centre Ecotox (2023). Proposals for Quality Criteria for Surface Waters: Oekotoxzentrum Centre Ecotox. https://www.ecotoxcentre.ch/expert-service/quality-criteria/quality-criteria-for-surface-waters?_ga=2.178848749.890633271.1680527698-1146943795.1680527698.

[5] (2019). Division protection des eaux (DGE-PRE) Section épuration urbaine. Micropolluants Dans les Stations D'Épuration Vaudoises.

[6] Li W.C. (2014). Occurrence, sources, and fate of pharmaceuticals in aquatic environment and soil. Environ. Pollut.

[7] Mackuľak T., Černanský S., Fehér M., Birošová L., Gál M. (2019). Pharmaceuticals, drugs, and resistant microorganisms—Environmental impact on population health. Curr. Opin. Environ. Sci. Health.

[8] Liu J.-L., Wong M.-H. (2013). Pharmaceuticals and personal care products (PPCPs): A review on environmental contamination in China. Environ. Int..

[9] Carter L.J., Williams M., Martin S., Kamaludeen S.P.B., Kookana R.S. (2018). Sorption, plant uptake and metabolism of benzodiazepines. Sci. Total Environ..

[10] Richmond E.K., Rosi E.J., Walters D.M., Fick J., Hamilton S.K., Brodin T., Sundelin A., Grace M.R. (2018). A diverse suite of pharmaceuticals contaminates stream and riparian food webs. Nat. Commun..

[11] Galus M., Jeyaranjaan J., Smith E., Li H., Metcalfe C., Wilson J.Y. (2013). Chronic effects of exposure to a pharmaceutical mixture and municipal wastewater in zebrafish. Aquat. Toxicol..

[12] Klatte S., Schaefer H.-C., Hempel M. (2017). Pharmaceuticals in the environment—A short review on options to minimize the exposure of humans, animals and ecosystems. Sustain. Chem. Pharm..

[13] Chaturvedi P., Shukla P., Giri B.S., Chowdhary P., Chandra R., Gupta P., Pandey A. (2021). Prevalence and hazardous impact of pharmaceutical and personal care products and antibiotics in environment: A review on emerging contaminants. Environ. Res.

[14] Zhang X., Yan S., Chen J., Tyagi R.D., Li J., Tyagi R.D., Sellamuthu B., Tiwari B., Yan S., Drogui P., Zhang X., Pandey A. (2020). 3—Physical, chemical, and biological impact (hazard) of hospital wastewater on environment: Presence of pharmaceuticals, pathogens, and antibiotic-resistance genes. Current Developments in Biotechnology and Bioengineering.

[15] Guideline on the Environmental Risk Assessment of Medicinal Products for Human Use, (2006). https://www.ema.europa.eu/en/documents/scientific-guideline/guideline-environmental-risk-assessment-medicinal-products-human-use-first-version_en.pdf.

[16] Stenuick J.Y. (2022). Recommendations for Greener Human Medicines in the Revision of the EU General Pharmaceuticals Legislation. https://europe.noharm.org/sites/default/files/documents-files/7065/2022-04-14_Position-paper_Recommendations-for-greener-medicines.pdf.

[17] Swiss Federal Council (2000). Ordonnance Sur Les Paiements Directs Versés Dans L’Agriculture (Ordonnance Sur Les Paiements Directs, OPD).

[18] Tennison I., Roschnik S., Ashby B., Boyd R., Hamilton I., Oreszczyn T., Owen A., Romanello M., Ruyssevelt P., Sherman J.D. (2021). Health care’s response to climate change: A carbon footprint assessment of the NHS in England. Lancet Planet Health.

[19] Royal College of General Practitioners Climate Change and Sustainability: Royal College of General Practitioners. https://www.rcgp.org.uk/policy/rcgp-policy-areas/climate-change-sustainable-development-and-health.

[20] Brooks S., Cheung A., Wintemute K. Low Carbon Inhalers: Choosing Wisely for Patients and the Environment. https://www.cfp.ca/news/2020/08/25/08-24.

[21] Adeyeye E., New B.J.M., Chen F., Kulkarni S., Fisk M., Coleman J.J. (2022). Sustainable medicines use in clinical practice: A clinical pharmacological view on eco-pharmaco-stewardship. Br. J. Clin. Pharmacol..

[22] Castensson S., Eriksson V., Lindborg K., Wettermark B. (2009). A method to include the environmental hazard in drug prescribing. Pharm World Sci.

[23] Håkonsen H., Dohle S., Rhedin H., Hedenrud T. (2023). Preferences for medicines with different environmental impact—A Swedish population-based study. Environ. Adv..

[24] (2021). Commission internationale pour la protection des eaux du Léman. Rapport Sur Les Études et Recherches Entreprises Dans le Bassin Lémanique—Campagne 2021.

[25] Stratégie de Surveillance et de Protection de la Qualité des Eaux Superficielles. https://www.vd.ch/fileadmin/user_upload/themes/environnement/eau/fichiers_pdf/DIREV_PRE/Strategie-Eau-web-doublepage.pdf.

[26] (2023). CIPEL—Commission internationale pour la protection des eaux du Léman. Eawag_Résultats_CIPEL_LCMS_LacLeman2021.

[27] Alliance A.I. (2023). AMR Alliance Science-Based PNEC Targets for Risk Assessments. AMR Industry Alliance. https://www.amrindustryalliance.org/wp-content/uploads/2023/02/AMR-Table-1-Update-20230222_corrected.pdf.

[28] (2025). NORMAN Ecotoxicology Database—Lowest PNECs [Internet]. THE NORMAN NETWORK. https://www.norman-network.com/nds/ecotox/lowestPnecsIndex.php.

[29] Federal Office for the Environment FOEN (2023). Micropollutants in the National Monitoring Programme: Swiss Confederation. https://www.bafu.admin.ch/bafu/en/home/topics/water/info-specialists/state-of-waterbodies/state-of-watercourses/water-quality-in-watercourses/micropollutants-in-watercourses.html#-542911050.

[30] European Union (2022). DÉCISION D’EXÉCUTION (UE) 2022/1307 DE LA COMMISSION du 22 Juillet 2022 Établissant Une Liste de Vigilance Relative Aux Substances Soumises à Surveillance à L’Échelle de L’Union Dans le Domaine de la Politique de L’Eau en Vertu de la Directive 2008/105/CE du Parlement Européen et du Conseil.

[31] Council S.F. Swiss Legislation on Water Quality. https://www.fedlex.admin.ch/eli/cc/1998/2863_2863_2863/fr.

[32] Niemi L., Landová P., Taggart M., Boyd K., Zhang Z., Gibb S. (2022). Spatiotemporal trends and annual fluxes of pharmaceuticals in a Scottish priority catchment. Environ. Pollut.

[33] Fonseca E., Hernández F., Ibáñez M., Rico A., Pitarch E., Bijlsma L. (2020). Occurrence and ecological risks of pharmaceuticals in a Mediterranean river in Eastern Spain. Environ. Int..

[34] Region Stockholm (2016). Janusinfo Pharmaceuticals and Environment Preface: Region Stockholm. https://www.janusinfo.se/beslutsstod/lakemedelochmiljo/pharmaceuticalsandenvironment/environment/preface.5.7b57ecc216251fae474882c8.html.

[35] Ramström H., Martini S., Borgendahl J., Ågerstrand M., Lärfars G., Ovesjö M.L. (2020). Pharmaceuticals and Environment: A web-based decision support for considering environmental aspects of medicines in use. Eur. J. Clin. Pharmacol..

[36] (2018). Tableau Récapitulatif des NQE Réglementaires et Propositions de VGE de l'INERIS [Internet]. INERIS. https://substances.ineris.fr/fr/page/9.

[37] Miller T.H., Bury N.R., Owen S.F., MacRae J.I., Barron L.P. (2018). A review of the pharmaceutical exposome in aquatic fauna. Environ. Pollut..

[38] Cussans A., Harvey G., Kemple T., Tomson M. (2021). Interventions to Reduce the Environmental Impact of Medicines: A UK perspective✰. J. Clim. Change Health.

[39] Osorio V., Marcé R., Pérez S., Ginebreda A., Cortina J.L., Barceló D. (2012). Occurrence and modeling of pharmaceuticals on a sewage-impacted Mediterranean river and their dynamics under different hydrological conditions. Sci. Total Environ..

[40] Li Y., Taggart M.A., McKenzie C., Zhang Z., Lu Y., Pap S., Gibb S.W. (2021). A SPE-HPLC-MS/MS method for the simultaneous determination of prioritised pharmaceuticals and EDCs with high environmental risk potential in freshwater. J. Environ. Sci..

